# Ethyl 5-methyl­imidazo[1,2-*a*]pyridine-2-carboxyl­ate

**DOI:** 10.1107/S1600536810026577

**Published:** 2010-07-14

**Authors:** Jin-hua Yao, Lan-fang Wang, Bing Guo, Kang An, Jian-ning Guan

**Affiliations:** a Department of Applied Chemistry, College of Science, Nanjing University of Technology, No.5 Xinmofan Road, Nanjing, Nanjing 210009, People’s Republic of China

## Abstract

The title compound, C_11_H_12_N_2_O_2_, was synthesized from the reaction of 6-methyl­pyridin-2-amine and ethyl 3-bromo-2-oxopropionate. In the mol­ecular structure, the six- and five-membered rings are individually almost planar with r.m.s. deviations of 0.003 and 0.002 Å, respectively. The two rings are almost coplanar, the dihedral angle between their planes being 1.4 (3)°. Inter­molecular C—H⋯O and C—H⋯N hydrogen bonds are present in the crystal structure.

## Related literature

For the biological properties of related compounds, see: Xia *et al.* (2005 ▶); Warshakoon *et al.* (2006 ▶); Imaeda *et al.* (2008 ▶). For the synthetic procedure, see: Xia *et al.* (2005 ▶). For bond-length data, see: Allen *et al.* (1987 ▶).
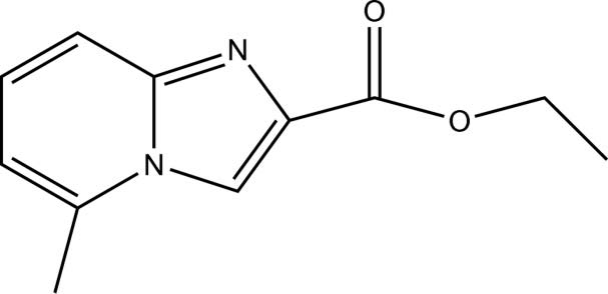


## Experimental

### 

#### Crystal data

C_11_H_12_N_2_O_2_
*M* *_r_* = 204.23
Monoclinic, 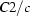

*a* = 17.164 (3) Å
*b* = 10.521 (2) Å
*c* = 13.759 (3) Å
β = 124.77 (3)°
*V* = 2041 (1) Å^3^
*Z* = 8
Mo *K*α radiation
μ = 0.09 mm^−1^
*T* = 293 K
0.30 × 0.20 × 0.10 mm

#### Data collection

Enraf–Nonius CAD-4 diffractometer
Absorption correction: ψ scan (North *et al.*, 1968 ▶) *T*_min_ = 0.973, *T*_max_ = 0.991
1923 measured reflections
1859 independent reflections
1360 reflections with *I* > 2σ(*I*)
*R*_int_ = 0.028
3 standard reflections every 200 reflections  intensity decay: 1%

#### Refinement

*R*[*F* ^2^ > 2σ(*F* ^2^)] = 0.051
*wR*(*F* ^2^) = 0.152
*S* = 1.00
1859 reflections
137 parameters
H-atom parameters constrained
Δρ_max_ = 0.24 e Å^−3^
Δρ_min_ = −0.20 e Å^−3^

### 

Data collection: *CAD-4 EXPRESS* (Enraf–Nonius, 1994 ▶); cell refinement: *CAD-4 EXPRESS*; data reduction: *XCAD4* (Harms & Wocadlo, 1995 ▶); program(s) used to solve structure: *SHELXS97* (Sheldrick, 2008 ▶); program(s) used to refine structure: *SHELXL97* (Sheldrick, 2008 ▶); molecular graphics: *SHELXTL* (Sheldrick, 2008 ▶); software used to prepare material for publication: *SHELXL97*.

## Supplementary Material

Crystal structure: contains datablocks global, I. DOI: 10.1107/S1600536810026577/im2214sup1.cif

Structure factors: contains datablocks I. DOI: 10.1107/S1600536810026577/im2214Isup2.hkl

Additional supplementary materials:  crystallographic information; 3D view; checkCIF report

## Figures and Tables

**Table 1:** Hydrogen-bond geometry (Å, °)

*D*—H⋯*A*	*D*—H	H⋯*A*	*D*⋯*A*	*D*—H⋯*A*
C2—H2*A*⋯N1^i^	0.93	2.59	3.461 (4)	155
C3—H3*A*⋯O1^i^	0.93	2.58	3.456 (4)	157

Symmetry code: (i) 
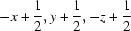
.

**Fig. 1.**
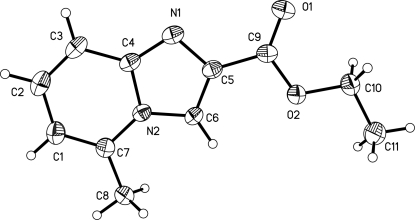
A view of the molecular structure of (I). Displacement ellipsoids are drawn at the 50% probability level.

**Fig. 2.**
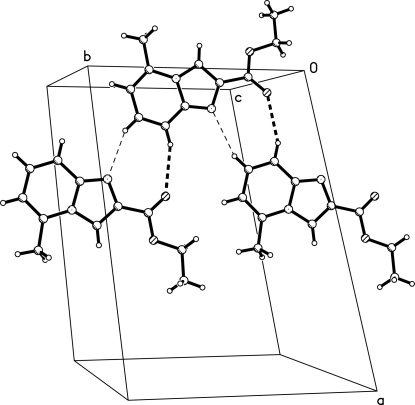
A packing diagram for (I). Dashed lines indicate an intermolecular C—H···N hydrogen bond and an intermolecular C—H···O hydrogen bond.

## supplementary crystallographic information

### Comment

Imidazo[1,2a]pyridine derivatives are of great interest because of their chemical and pharmaceutical properties. Some derivatives play a key role in preparing hypoxia inducible factor 1α prolyl hydroxylase inhibitors. And HIF-1α is of great potential value for treating ischemic diseases (Warshakoon *et al.*,2006). Some can be used as a material for preparing a series of FXa inhibitors, which can be used to cure various kinds of thromboembolic diseases (Imaeda *et al.*,2008). Herein we report the crystal structure of the title compound, (I). The molecular structure of (I) is shown in Fig.1. In this structure ring A (C1/C2/C3/C4/N2/C7) is a planar six-mermbered ring and the mean deviation from plane is 0.0027 Å, ring B(C4/N1/C5/C6/N2) is a planar five-mermbered ring with a mean deviation from planarity of 0.0018 Å. The dihedral angle between A and B ring is 1.4 (3)°. In the crystal structure, intermolecular C—H···O and C—H···N hydrogen bonds (Table 1) link the molecules to form a trimeric unit (Fig. 2).

### Experimental

6-Methyl-pyridin-2-ylamine (8 mmol) and 3-bromo-2-oxo-propionic acid ethyl ester (12 mmol) were added to 50 mL ethanol. The mixture was refluxed for 6 h and then the solvent was totally evaporated. Solid anhydrous KHCO_3_ was added until pH=8 had been reached. Set aside for 3 h led to the observation of a white, flocculent precipitate, which was filtered and dried (yield ca. 52.6%, Xia *et al.* (2005)). Crystals suitable for X-ray analysis were obtained by dissolving (I) (0.5 g) in ethyl acetate (20 ml) and evaporating the solvent slowly at room temperature for about 10 d.

### Refinement

All H atoms were positioned geometrically, with C—H= 0.97, 0.96 and 0.93 Å for methylene, methyl and aromatic H atoms, respectively, and constrained to ride on their parent atoms, with *U*_iso_(H)=*xU*_eq_(C), where *x*=1.5 for methyl H atoms and *x*=1.2 for all other H atoms.

### Crystal data

**Table:**

C_11_H_12_N_2_O_2_	*F*(000) = 864
*M**_r_* = 204.23	*D*_x_ = 1.329 Mg m^−^^3^
Monoclinic, *C*2/*c*	Melting point = 425–426 K
Hall symbol: -C 2yc	Mo *K*α radiation, λ = 0.71073 Å
*a* = 17.164 (3) Å	Cell parameters from 25 reflections
*b* = 10.521 (2) Å	θ = 9–13°
*c* = 13.759 (3) Å	µ = 0.09 mm^−^^1^
β = 124.77 (3)°	*T* = 293 K
*V* = 2041 (1) Å^3^	Block, colorless
*Z* = 8	0.30 × 0.20 × 0.10 mm

### Data collection

**Table:**

Enraf–Nonius CAD-4 diffractometer	1360 reflections with *I* > 2σ(*I*)
Radiation source: fine-focus sealed tube	*R*_int_ = 0.028
graphite	θ_max_ = 25.3°, θ_min_ = 2.4°
ω/2θ scans	*h* = −20→0
Absorption correction: ψ scan (North *et al.*, 1968)	*k* = −12→0
*T*_min_ = 0.973, *T*_max_ = 0.991	*l* = −13→16
1923 measured reflections	3 standard reflections every 200 reflections
1859 independent reflections	intensity decay: 1%

### Refinement

**Table:**

Refinement on *F*^2^	Secondary atom site location: difference Fourier map
Least-squares matrix: full	Hydrogen site location: inferred from neighbouring sites
*R*[*F*^2^ > 2σ(*F*^2^)] = 0.051	H-atom parameters constrained
*wR*(*F*^2^) = 0.152	*w* = 1/[σ^2^(*F*_o_^2^) + (0.1*P*)^2^] where *P* = (*F*_o_^2^ + 2*F*_c_^2^)/3
*S* = 1.00	(Δ/σ)_max_ < 0.001
1859 reflections	Δρ_max_ = 0.24 e Å^−^^3^
137 parameters	Δρ_min_ = −0.20 e Å^−^^3^
0 restraints	Extinction correction: *SHELXL97* (Sheldrick, 2008), Fc^*^=kFc[1+0.001xFc^2^λ^3^/sin(2θ)]^-1/4^
Primary atom site location: structure-invariant direct methods	Extinction coefficient: 0.0117 (17)

### Special details

**Table:**

Geometry. All e.s.d.'s (except the e.s.d. in the dihedral angle between two l.s. planes) are estimated using the full covariance matrix. The cell e.s.d.'s are taken into account individually in the estimation of e.s.d.'s in distances, angles and torsion angles; correlations between e.s.d.'s in cell parameters are only used when they are defined by crystal symmetry. An approximate (isotropic) treatment of cell e.s.d.'s is used for estimating e.s.d.'s involving l.s. planes.
Refinement. Refinement of *F*^2^ against ALL reflections. The weighted *R*-factor *wR* and goodness of fit *S* are based on *F*^2^, conventional *R*-factors *R* are based on *F*, with *F* set to zero for negative *F*^2^. The threshold expression of *F*^2^ > σ(*F*^2^) is used only for calculating *R*-factors(gt) *etc*. and is not relevant to the choice of reflections for refinement. *R*-factors based on *F*^2^ are statistically about twice as large as those based on *F*, and *R*- factors based on ALL data will be even larger.

### Fractional atomic coordinates and isotropic or equivalent isotropic displacement parameters (Å^2^)

**Table:**

	*x*	*y*	*z*	*U*_iso_*/*U*_eq_	
N1	0.12866 (11)	0.40243 (18)	0.19751 (15)	0.0466 (5)	
O1	0.07064 (11)	0.13738 (15)	0.15389 (15)	0.0602 (5)	
C1	0.06679 (17)	0.7803 (2)	0.15626 (19)	0.0523 (6)	
H1B	0.0502	0.8658	0.1452	0.063*	
N2	0.02673 (11)	0.56505 (15)	0.13920 (13)	0.0376 (4)	
O2	−0.07427 (10)	0.20448 (14)	0.09371 (13)	0.0512 (5)	
C2	0.16246 (17)	0.7466 (3)	0.2109 (2)	0.0582 (6)	
H2A	0.2075	0.8102	0.2346	0.070*	
C3	0.19016 (14)	0.6232 (2)	0.22978 (18)	0.0527 (6)	
H3A	0.2537	0.6020	0.2665	0.063*	
C4	0.12108 (13)	0.5273 (2)	0.19286 (17)	0.0422 (5)	
C5	0.03813 (14)	0.3590 (2)	0.14702 (17)	0.0408 (5)	
C6	−0.02457 (13)	0.45553 (19)	0.11134 (16)	0.0387 (5)	
H6A	−0.0896	0.4487	0.0751	0.046*	
C7	−0.00174 (15)	0.6916 (2)	0.11927 (17)	0.0432 (5)	
C8	−0.10473 (15)	0.7154 (2)	0.05724 (19)	0.0509 (6)	
H8A	−0.1164	0.8053	0.0480	0.076*	
H8B	−0.1260	0.6807	0.1028	0.076*	
H8C	−0.1385	0.6757	−0.0192	0.076*	
C9	0.01645 (14)	0.2218 (2)	0.13352 (17)	0.0432 (5)	
C10	−0.10735 (16)	0.0748 (2)	0.0699 (2)	0.0560 (6)	
H10A	−0.0701	0.0241	0.1412	0.067*	
H10B	−0.1011	0.0386	0.0098	0.067*	
C11	−0.20816 (17)	0.0755 (3)	0.0283 (2)	0.0727 (8)	
H11A	−0.2321	−0.0099	0.0114	0.109*	
H11B	−0.2443	0.1261	−0.0421	0.109*	
H11C	−0.2134	0.1109	0.0887	0.109*	

### Atomic displacement parameters (Å^2^)

**Table:**

	*U*^11^	*U*^22^	*U*^33^	*U*^12^	*U*^13^	*U*^23^
N1	0.0347 (9)	0.0568 (12)	0.0450 (10)	0.0026 (8)	0.0208 (8)	0.0007 (8)
O1	0.0514 (10)	0.0524 (10)	0.0734 (11)	0.0127 (8)	0.0335 (9)	0.0054 (8)
C1	0.0589 (14)	0.0472 (13)	0.0525 (13)	−0.0098 (11)	0.0328 (11)	−0.0024 (10)
N2	0.0335 (9)	0.0460 (10)	0.0347 (9)	−0.0019 (7)	0.0202 (7)	−0.0025 (7)
O2	0.0443 (9)	0.0417 (9)	0.0654 (10)	−0.0026 (6)	0.0301 (8)	−0.0048 (7)
C2	0.0540 (14)	0.0666 (16)	0.0554 (13)	−0.0196 (12)	0.0320 (11)	−0.0058 (12)
C3	0.0362 (11)	0.0717 (17)	0.0488 (13)	−0.0106 (10)	0.0234 (10)	−0.0027 (11)
C4	0.0330 (10)	0.0564 (14)	0.0360 (10)	−0.0003 (9)	0.0188 (8)	−0.0004 (9)
C5	0.0365 (10)	0.0492 (12)	0.0365 (10)	0.0012 (9)	0.0207 (9)	0.0003 (9)
C6	0.0327 (10)	0.0457 (12)	0.0372 (10)	−0.0037 (9)	0.0196 (8)	−0.0020 (9)
C7	0.0474 (12)	0.0466 (13)	0.0387 (11)	−0.0031 (10)	0.0264 (9)	−0.0024 (9)
C8	0.0498 (13)	0.0447 (13)	0.0588 (13)	0.0022 (10)	0.0313 (11)	−0.0019 (10)
C9	0.0397 (11)	0.0491 (13)	0.0401 (11)	0.0056 (9)	0.0223 (9)	0.0022 (9)
C10	0.0557 (14)	0.0427 (13)	0.0662 (15)	−0.0034 (10)	0.0327 (12)	−0.0038 (11)
C11	0.0557 (15)	0.0608 (17)	0.092 (2)	−0.0087 (12)	0.0369 (15)	−0.0109 (14)

### Geometric parameters (Å, °)

**Table:**

N1—C4	1.318 (3)	C3—H3A	0.9300
N1—C5	1.369 (2)	C5—C6	1.352 (3)
O1—C9	1.198 (2)	C5—C9	1.475 (3)
C1—C7	1.353 (3)	C6—H6A	0.9300
C1—C2	1.407 (3)	C7—C8	1.482 (3)
C1—H1B	0.9300	C8—H8A	0.9600
N2—C6	1.366 (2)	C8—H8B	0.9600
N2—C7	1.391 (3)	C8—H8C	0.9600
N2—C4	1.401 (2)	C10—C11	1.480 (3)
O2—C9	1.336 (2)	C10—H10A	0.9700
O2—C10	1.442 (3)	C10—H10B	0.9700
C2—C3	1.357 (4)	C11—H11A	0.9600
C2—H2A	0.9300	C11—H11B	0.9600
C3—C4	1.412 (3)	C11—H11C	0.9600
			
C4—N1—C5	104.84 (16)	C1—C7—C8	126.6 (2)
C7—C1—C2	121.8 (2)	N2—C7—C8	116.43 (18)
C7—C1—H1B	119.1	C7—C8—H8A	109.5
C2—C1—H1B	119.1	C7—C8—H8B	109.5
C6—N2—C7	130.90 (17)	H8A—C8—H8B	109.5
C6—N2—C4	105.97 (16)	C7—C8—H8C	109.5
C7—N2—C4	123.11 (16)	H8A—C8—H8C	109.5
C9—O2—C10	116.01 (17)	H8B—C8—H8C	109.5
C3—C2—C1	121.2 (2)	O1—C9—O2	124.2 (2)
C3—C2—H2A	119.4	O1—C9—C5	126.1 (2)
C1—C2—H2A	119.4	O2—C9—C5	109.67 (17)
C2—C3—C4	119.0 (2)	O2—C10—C11	107.8 (2)
C2—C3—H3A	120.5	O2—C10—H10A	110.2
C4—C3—H3A	120.5	C11—C10—H10A	110.2
N1—C4—N2	111.15 (17)	O2—C10—H10B	110.2
N1—C4—C3	130.93 (19)	C11—C10—H10B	110.2
N2—C4—C3	117.91 (19)	H10A—C10—H10B	108.5
C6—C5—N1	111.79 (19)	C10—C11—H11A	109.5
C6—C5—C9	126.71 (19)	C10—C11—H11B	109.5
N1—C5—C9	121.48 (18)	H11A—C11—H11B	109.5
C5—C6—N2	106.24 (17)	C10—C11—H11C	109.5
C5—C6—H6A	126.9	H11A—C11—H11C	109.5
N2—C6—H6A	126.9	H11B—C11—H11C	109.5
C1—C7—N2	117.0 (2)		
			
C7—C1—C2—C3	−0.6 (3)	C4—N2—C6—C5	−0.42 (19)
C1—C2—C3—C4	0.4 (3)	C2—C1—C7—N2	0.9 (3)
C5—N1—C4—N2	−0.5 (2)	C2—C1—C7—C8	−177.8 (2)
C5—N1—C4—C3	−179.0 (2)	C6—N2—C7—C1	−178.96 (19)
C6—N2—C4—N1	0.6 (2)	C4—N2—C7—C1	−1.2 (3)
C7—N2—C4—N1	−177.71 (17)	C6—N2—C7—C8	−0.2 (3)
C6—N2—C4—C3	179.30 (17)	C4—N2—C7—C8	177.64 (16)
C7—N2—C4—C3	1.0 (3)	C10—O2—C9—O1	−2.8 (3)
C2—C3—C4—N1	177.8 (2)	C10—O2—C9—C5	176.75 (17)
C2—C3—C4—N2	−0.6 (3)	C6—C5—C9—O1	171.5 (2)
C4—N1—C5—C6	0.2 (2)	N1—C5—C9—O1	−6.9 (3)
C4—N1—C5—C9	178.87 (19)	C6—C5—C9—O2	−8.0 (3)
N1—C5—C6—N2	0.2 (2)	N1—C5—C9—O2	173.49 (16)
C9—C5—C6—N2	−178.43 (18)	C9—O2—C10—C11	179.40 (18)
C7—N2—C6—C5	177.67 (18)		

### Hydrogen-bond geometry (Å, °)

**Table:**

*D*—H···*A*	*D*—H	H···*A*	*D*···*A*	*D*—H···*A*
C2—H2A···N1^i^	0.93	2.59	3.461 (4)	155
C3—H3A···O1^i^	0.93	2.58	3.456 (4)	157

Symmetry codes: (i) −*x*+1/2, *y*+1/2, −*z*+1/2.
